# Quantitative Perfusion Assessment Using Indocyanine Green in Lower Extremity Perforator Flaps

**DOI:** 10.1177/15533506251339929

**Published:** 2025-05-19

**Authors:** Lasse W. P. Van ’t Hof, Isabelle T. S. Koster, Richard M. Van den Elzen, Mark-Bram Bouman, Matthijs Botman, Caroline Driessen

**Affiliations:** 1 Department of Plastic, Reconstructive and Hand surgery, Amsterdam University Medical Center, Amsterdam, The Netherlands; 2 Amsterdam Movement Sciences, Amsterdam University Medical Center, Amsterdam, The Netherlands; 3 Cancer Center Amsterdam, Imaging and Biomarkers, Amsterdam, The Netherlands; 4 Biomedical Engineering and Physics, 571151 Amsterdam UMC Location University of Amsterdam, Amsterdam, The Netherlands

**Keywords:** fluorescence imaging, indocyanine green, lower extremity reconstruction

## Abstract

**Background:**

Indocyanine-Green Fluorescence Angiography (ICG-FA) is widely used in reconstructive surgery, providing real-time visualization of flap perfusion. Accurate assessment of perfusion is especially critical in lower extremity reconstructions, where complications like necrosis and venous congestion can lead to poor outcomes, including amputation. Although ICG-FA is commonly available, its interpretation remains subjective and heavily reliant on the surgeon’s experience. These challenges underline the importance of integrating objective, data-driven assessment tools into surgical practice.

**Methods:**

As part of a larger, ongoing prospective study, three illustrative cases of lower extremity reconstructions using perforator-based fasciocutaneous flaps were selected. Intraoperative ICG-FA was performed using a surgical microscope with integrated fluorescence imaging. Fluorescence-time-curves (FTCs) were generated using specialized software, and associated quantitative perfusion parameters were compared across three cases: two patients with perfusion-related complications and one patient without complications.

**Results:**

Intraoperative clinical assessment appeared satisfactory in all cases, and no changes in surgical management were made based on the subjective interpretation of ICG-FA. In contrast, quantitative analysis of ICG-FA revealed abnormal perfusion patterns in the two flaps that developed complications, identifying perfusion deficits not evident through conventional assessment.

**Conclusion:**

These findings suggest that FTCs derived from ICG-FA data can predict perfusion-related complications. Integrating quantitative ICG-FA analysis into clinical practice may yield a significant advancement in reconstructive surgery, especially in lower extremity reconstructions.

**Clinical trial name:**

ICG Indocyanine Green in Reconstructive Surgery (ICG-R).

**ClinicalTrials.gov ID:**

NCT06129669 (https://clinicaltrials.gov/study/NCT06129669?cond=NCT06129669&rank=1).

## Introduction

A thorough assessment of perfusion is vital for the successful outcome of lower leg reconstructions, especially in traumatized legs where vascular compromise may occur if reconstruction does not extend well above the zone of injury. Factors such as vessel kinking, suboptimal flap design, or improper inset can significantly increase the risk of inadequate perfusion, leading to complications such as necrosis and venous congestion. This can compromise flap survival and, in severe cases, result in limb amputation.^1,2^ Currently, flap viability is evaluated through clinical indicators such as capillary refill, temperature, color and wound edge bleeding. Despite the expertise of reconstructive surgeons, complication rates for lower leg perforator-based flaps remain high, ranging from 26-42%, underscoring the need for more reliable and precise tools for intraoperative perfusion assessment.^3,4^

In addition to clinical evaluation, a variety of perioperative methods exist for assessing flap perfusion, including Doppler ultrasonography, oxygen saturation monitoring, dynamic infrared thermography, and fluorescence imaging. Among these, Indocyanine-Green Fluorescence Angiography (ICG-FA) has emerged as a powerful tool for real-time visualization of tissue perfusion and has been widely investigated and adopted in various surgical fields, including colorectal, vascular, gastrointestinal, and reconstructive surgery.^5-7^ In these contexts, ICG-FA has been used to guide intraoperative decision-making and has been associated with reduced complication rates. While traditionally interpreted subjectively, integrating quantitative analysis into ICG-FA is increasingly explored across specialties, offering the potential to provide objective perfusion metrics.^8^ This innovation could represent a more accurate method for assessing perfusion and identifying lower extremity flaps at risk for complications.

Despite these advances, the application of ICG-FA in lower extremity reconstruction remains limited.^6,9^ In many centers, the technique is still infrequently used during these procedures, often due to unfamiliarity with the equipment or the need for additional setup. A potential solution lies in the use of the surgical microscope, which is routinely employed in microsurgical lower extremity reconstructions and is therefore already available in the operating room. Many of these microscopes feature an integrated fluorescence mode, making them a practical and accessible platform for ICG-FA without the need for separate imaging systems.^10^

In this study, we present three illustrative cases from an ongoing prospective series, comparing quantitative ICG-FA parameters in lower extremity perforator-based flaps with and without postoperative complications. The aim was to show the benefit of quantification of ICG-FA to predict adverse outcomes more accurately than clinical or subjective ICG-FA assessment alone.

## Methods

### Study Design

This study is part of an ongoing prospective cohort investigating quantitative perfusion assessment in reconstructive surgery using ICG-FA. From this larger cohort, three representative cases were selected for detailed analysis in this report. These cases illustrate three distinctly different clinical outcomes and their corresponding perfusion patterns: (1) uncomplicated healing, (2) venous insufficiency, and (3) arterial insufficiency.

### Surgical Procedure and Perfusion Assessment

All patients underwent reconstruction of the lower extremity using pedicled perforator-based fasciocutaneous flaps. Clinical evaluation of flap perfusion was performed intraoperatively and included assessment of capillary refill, skin color, temperature, and wound edge bleeding.

Following clinical assessment, ICG-FA was conducted in a standardized manner using intravenously administered Indocyanine Green (IC-Green™, Akorn Pharmaceuticals, Lake Forest, IL, USA) and the near-infrared function (IR-800) of the Zeiss Tivato 700 surgical microscope (Carl Zeiss Meditec AG, Oberkochen, Germany). A snapshot of the field of view was captured using the microscope to enable postoperative correlation between clinical outcomes and the ICG-FA data. Given the limited field of view of a surgical microscope, the regions of the flap which are most prone to perfusion-related complications were selected for imaging.

### Quantitative Fluorescence Analysis

Postoperatively, the raw ICG-FA data were analyzed using custom software, which involved positioning three regions of interest (ROIs): ROI 1 (red) in a well-vascularized reference area outside of the flap, ROI 2 (blue) proximally within the flap and ROI 3 (yellow) distally within the flap. This analysis generates fluorescence-time-curves (FTCs), presented as both absolute and normalized curves. For normalization, the maximum fluorescence intensity (Fmax) in the selected ROI is set to 100%, and fluorescence over time is expressed as a percentage of Fmax. This approach minimizes case-specific variability and facilitates direct comparisons across ROIs.^11^ Quantitative parameters such as inflow slope (ingress), outflow slope (egress), and time to peak fluorescence (Ttp) were derived and compared across ROIs and cases. ROI 1 served as a reference curve for adequate perfusion, characterized by steep inflow and steady outflow.

### Postoperative Evaluation

Postoperative follow-up was conducted for up to six weeks to assess flap outcomes. Complications such as necrosis or signs of venous congestion were recorded and compared with intraoperative FTC patterns and quantitative perfusion parameters.

## Results

### Case 1 – No Complication

**Figure 1.**
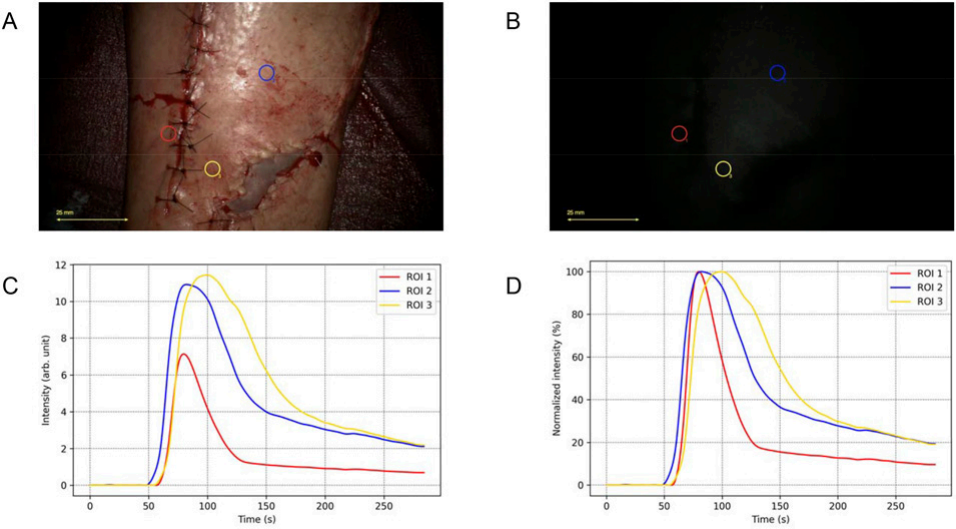
Fluorescence-time-curve (FTC) analysis for patient 1. (A) White-light image showing the placement of regions of interest (ROIs) for tissue perfusion analysis. Red indicates ROI 1 (normal vascularized reference area), blue indicates ROI 2 (proximal flap tissue), and yellow indicates ROI 3 (distal flap tissue). A split-thickness skin graft is visible on the most caudolateral side. (B) Near-infrared fluorescence signal following indocyanine green (ICG) administration. Minimal fluorescence intensity was observed, likely due to the microscope being positioned too far from the surgical site. (C) Absolute time-intensity curves showing fluorescence intensity over time for each ROI. The curves reveal consistently low absolute intensity across all regions of interest. (D) Normalized time-intensity curves, with fluorescence intensity scaled to a maximum of 100% (Fmax) for each ROI. The normalized curves highlight favorable inflow and outflow characteristics across the ROIs.

The first patient developed a pretibial defect on the leg following a deep wound infection involving osteosynthesis hardware, eight weeks after a crural fracture. During surgery, the orthopedic team removed the infected hardware and inserted a tibial nail. Subsequently, a local perforator-based transposition flap was used for coverage. Intraoperatively, clinical assessment, based on capillary refill, color, and temperature, confirmed satisfactory perfusion of the flap. ICG-FA revealed relatively low fluorescence intensity, likely related to suboptimal imaging conditions, but was not interpreted as suspicious. No perfusion-related complications were observed during the 6-week follow-up period. ICG-FA data were plotted into FTCs as shown in Figure 1. The FTC characteristics within the flap resembled those of well-vascularized tissue.

### Case 2 – Venous Insufficiency

**Figure 2.**
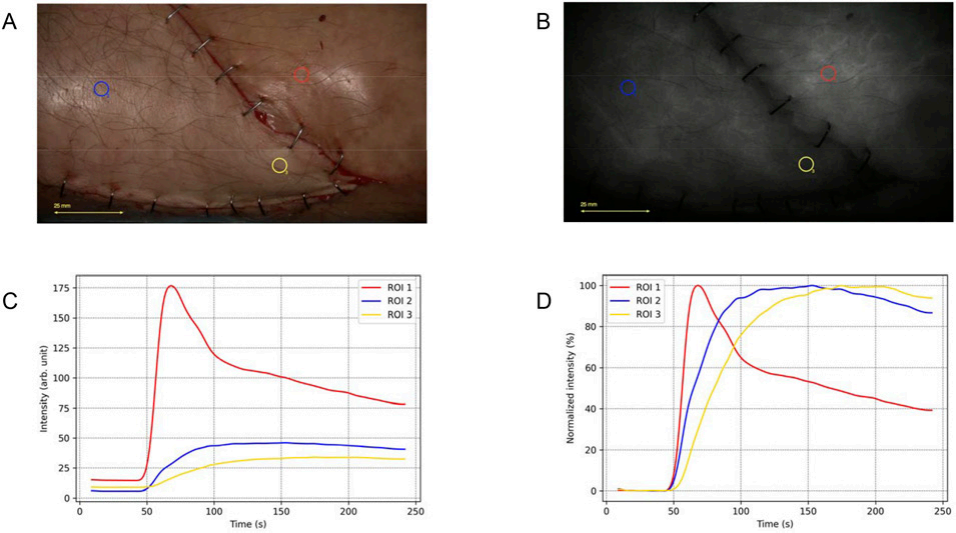
Fluorescence-time-curve (FTC) analysis for patient 2. (A) White-light image showing the placement of regions of interests (ROIs). (B) Near-infrared fluorescence assessment, showing adequate fluorescence intensity across the flap. (C) Absolute time-intensity curves revealing diminished absolute fluorescence intensity in the flap tissue compared to the normalized reference area, indicating reduced perfusion. (D) Normalized time-intensity curves demonstrating slow inflow and slow outflow characteristics within the flap tissue, suggesting impaired vascular dynamics.

The second patient had undergone hardware placement for an ankle fracture three years earlier, which was later removed due to discomfort. Four weeks later, a soft tissue defect developed from wound complications. We performed a propellor flap based on a perforator of the posterior tibial artery. After identifying a suitable perforator, the flap was rotated 180° without tension. Intraoperative clinical evaluation showed normal capillary refill, warm flap temperature, and adequate wound edge bleeding. Subjective ICG-FA also appeared satisfactory. However, two days postoperatively, signs of venous congestion emerged, necessitating re-operation. During the revision procedure, a more thorough dissection of the posterior tibial vessels improved venous outflow, and the patient recovered without further complications. Unfortunately, ICG-FA was not performed during the revision procedure. The FTCs for this case are shown in Figure 2 and reveal slow and limited inflow into the flap, followed by a plateau with minimal outflow.

### Case 3 – Arterial Insufficiency

**Figure 3.**
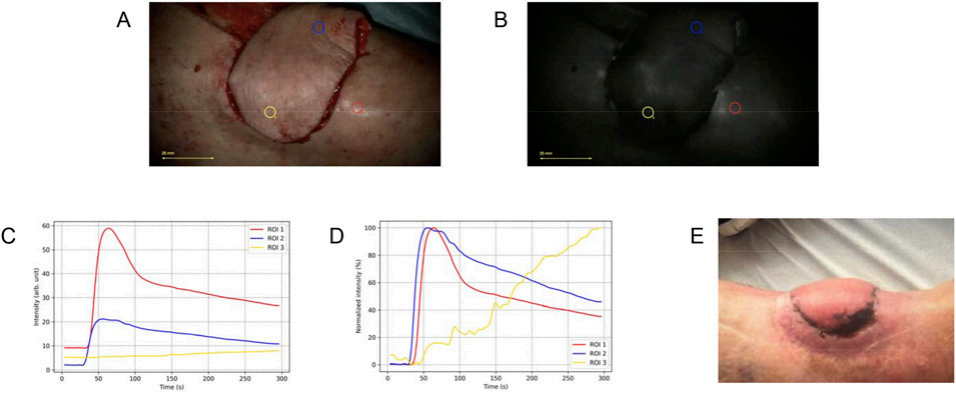
Fluorescence-time-curve (FTC) analysis for patient 3. (A) White-light image showing the placement of regions of interests (ROIs). (B) Near-infrared fluorescence assessment showing diminished fluorescence intensity in the distal region of the flap. (C) Absolute time-intensity curves revealing reduced fluorescence intensity within the flap tissue, particularly in the distal area. (D) Normalized time-intensity curves demonstrating very slow inflow characteristics and the absence of outflow in the distal region of the flap. (E) Clinical image showing superficial necrosis on the distal aspect of the flap.

The third patient developed a chronic pretibial defect after a dog bite. Surgical reconstruction was performed using a distally pedicled, perforator-based fasciocutaneous flap. Intraoperative clinical indicators, including capillary refill and distal wound edge bleeding, appeared adequate. Subsequently, ICG-FA was performed and revealed reduced fluorescence intensity in the distal portion of the flap. Although reduced fluorescence intensity was observed in the distal flap region, limited familiarity with ICG-FA interpretation contributed to the decision to proceed based on clinical indicators, which were judged to be adequate at the time. One week later, superficial necrosis was observed on the flap’s distal aspect, at the level of ROI 3. The FTCs and the postoperative clinical picture are shown in Figure 3. The FTCs reveal sufficient inflow and outflow in the proximal part of the flap, while almost no inflow was observed in the most distal area of the flap where necrosis occurred. We performed a debridement on the outpatient clinic after which the wound healed well by secondary intention.

### Quantitative Analysis

Quantitative parameters are listed in Table 1. In patient 1, fluorescence parameters were nearly comparable between the normal vascularized tissue and the flap tissue. In patient 2, who experienced venous congestion, we observed reduced ingress and, particularly, egress parameters. In patient 3, fluorescence parameters were comparable between the normal vascularized tissue and the flap tissue proximal to the pedicle. However, in the distal area where necrosis occurred, we observed significantly diminished ingress parameters and an absence of egress parameters.

**Table 1.** Quantitative Parameters of Perfusion in all Three Patients.

Fluorescence Parameter	Patient 1			Patient 2			Patient 3		
	No Complication			Venous Congestion			Distal Necrosis		
	Normal Vascularized Reference Area	Flap Tissue		Normal Vascularized Reference Area	Flap Tissue		Normal Vascularized Reference Area	Flap Tissue	
	ROI 1	ROI 2	ROI 3	ROI 1	ROI 2	ROI 3	ROI 1	ROI 2	ROI 3
Inflow parameters (Ingress)									
_Ttp_ (s)	18.0	27.2 (1.5)	38.6 (2.1)	22.9	105.3 (4.6)	121.8 (5.3)	28.4	24.7 (0.9)	291.8 (10.3)
SN_mean_inflow (%/s)_	5.6	3.6 (1.6)	2.6 (2.2)	3.1	0.9 (3.4)	0.8 (3.9)	3.1	3.5 (0.9)	0.3 (10.3)
SN_max_inflow (%/s)_	8.6	7.2 (1.2)	6.6 (1.3)	8.9	4.0 (2.2)	2.3 (3.9)	7.7	8.9 (0.9)	2.9 (2.7)
Outflow parameters (Egress)									
SN_mean_outflow (%/s)_	0.4	0.4 (1.0)	0.4 (1.0)	0.4	0.2 (2.0)	0.1 (4.0)	0.3	0.2 (1.5)	NM
SN_max_outflow (%/s)_	1.3	1.8 (0.7)	1.4 (0.9)	1.6	0.3 (5.3)	0.3 (5.3)	1.4	1.0 (1.4)	NM
T_90%_ (s)	7.4	20.3 (2.7)	17.2 (2.3)	18.3	79.0 (4.3)	NM	17.8	33.8 (1.9)	NM
T_80%_ (s)	11.5	26.4 (2.3)	29.8 (2.6)	27.24	NM	NM	27.0	55.3 (2.0)	NM

Derived from the fluorescence-time-curve (FTC), the software computes numerous quantitative perfusion parameters for each region of interest (ROI), including time from t0 to Fmax (_Ttp_), normalized mean slope inflow (SN_mean_inflow_), normalized maximum slope inflow (SN_max_inflow_), normalized mean slope outflow (SN_mean_outflow_), normalized maximum slope outflow (SN_max_outflow_), time from Fmax to 90% of Fmax (T_90%_), time from Fmax to 80% of Fmax (T_80%_). The normalized analysis measures fluorescence intensity over time as a percentage of the maximum recorded intensity. The normalized slope for the ingress and egress are defined as percentage per second (%/s). Between parentheses, relative values are shown. Relative values for time are calculated by dividing parameters in ROI 2 or 3 (flap tissue) by parameters in ROI 1 (normal vascularized tissue). Relative values for the normalized slope are calculated by dividing parameters in ROI 1 by parameters in ROI 2 or 3. NM: not measurable; no outflow detected.

The fluorescence images and corresponding FTCs for all three patients are shown in Supplemental Digital Content 1 (Video demonstrating ICG-FA and corresponding FTCs for all three patients).

## Discussion

This study demonstrates the potential of quantitative FTC analysis to detect perfusion differences in lower extremity flaps that are not reliably identified through clinical assessment or subjective interpretation of ICG-FA. Both clinical indicators and subjective interpretation of ICG-FA underestimated the severity of perfusion deficits. FTC analysis, however, revealed clear differences in inflow and outflow dynamics that correlated with clinical outcomes.

Despite its widespread use in other surgical fields, ICG-FA remains underutilized in lower extremity reconstruction.^6,12,13^ This limited adoption is likely driven by several factors, including the technical challenges of imaging the anatomically complex lower limb, inconsistent reproducibility of fluorescence signals, and the absence of standardized interpretation criteria. To support broader implementation of ICG-FA in this setting, the technique must be accessible, reproducible, and seamlessly integrated into existing surgical workflows. One practical solution that fulfills these requirements is the surgical microscope. Beyond its routine availability, it provides the advantage of enabling intraoperative fluorescence imaging without altering the surgical setup or requiring additional equipment. This low-threshold integration may help overcome logistical barriers and promote more widespread use of ICG-FA in reconstructive procedures. These cases show that even with a limited field of view, objective ICG-FA is an extremely useful adjunct to subjective clinical and fluorescence assessment. Clinical utility is still limited by this subjectivity of visual assessment of ICG-FA. In this study, for instance, lower fluorescence intensity was observed intraoperatively in a patient who later developed necrosis. However, no action was taken at the time due to favorable clinical indicators. This example illustrates a broader challenge in surgical practice. Without objective criteria, surgeons may be reluctant to act on the innovational fluorescence findings, particularly when image quality is suboptimal or when personal experience with the technique is limited.

Quantitative analysis of FTCs offers a promising solution to overcome the limitations of subjective interpretation. By providing objective, measurable parameters such as inflow slope, time to peak, and outflow dynamics, FTCs allow for a more reproducible and data-driven evaluation of flap perfusion. The method used in this study is based on analysis techniques that have already been successfully applied in other surgical fields in the Amsterdam UMC.^14,15^ In contrast to visual interpretation, which can be influenced by operator experience and variable image quality,^16^ FTCs remain clinically informative even in cases with low signal intensity. This was particularly evident in one case within our series, where despite weak overall fluorescence, the FTCs demonstrated normal perfusion patterns and the flap healed uneventfully. These findings support the potential role of FTC analysis as a complementary tool to enhance intraoperative decision-making.

Although these results are promising, the integration of FTC analysis into microscope-assisted fluorescence imaging workflows presents both opportunities and challenges. While the surgical microscope offered a convenient means to perform ICG-FA without additional equipment, it also introduced technical constraints. Its optical zoom system required a relatively large working distance to capture broader areas, which may have contributed to lower fluorescence intensity. Moreover, the limited field of view made it impossible to visualize the entire flap in a single image, necessitating targeted imaging of areas suspected to be at higher risk of complications.

The quantification method used in this study represents an earlier generation of fluorescence analysis.^11,14,15,17-20^ While more advanced techniques have since been developed,^21^ particularly in fields such as colorectal and breast surgery,^22^ where algorithm-driven software now enables real-time objective perfusion assessment, our approach remains valuable in a clinical context. Simpler quantification methods such as FTC analysis may provide an important bridge toward implementation by generating clear insights into perfusion dynamics and facilitating the development of clinically relevant cut-off values. Furthermore, universally accepted thresholds for key parameters such as inflow slope and time to peak are still lacking in the context of lower extremity reconstructions.

## Conclusion

This study demonstrates the feasibility of FTC analysis using microscope-integrated ICG-FA to assess perfusion in lower extremity flaps. The method revealed clear distinctions between arterial and venous insufficiency that were not apparent through conventional assessment alone. ICG-FA and FTC analysis have a high potential to support intraoperative decision-making in the complicated field of lower extremity reconstructions.

## Supplemental Material

**Figure:** Video 1. Indocyanine Green Fluorescence Angiography (ICG-FA) assessments and corresponding FTCs for all three patients.
